# Supplementary material for: Energy and Macronutrient Dietary Intakes of Vegetarian and Semi-Vegetarian Serbian Adults: Data from the EFSA EU Menu Food Consumption Survey (2017–2022)
Source: Foods. 2025 Apr 8;14(8):1285. doi: 10.3390/foods14081285 (PMC12027131; doi:10.3390/foods14081285)
Supplement: Supplementary file 1 [file foods-14-01285-s001.zip › foods-3481880-supplementary.pdf]

foods

MDPI

Article

Energy and macronutrient dietary intakes of the vegetarian and semi-vegetarian Serbian adults: data from the EFSA EU Menu Food Consumption Survey (2017-2022)

Supplementary material

Supplementary tables

Supplementary Table S1. Total daily energy intake from different food groups in a sample of vegetarians/semi-vegetarians 18-74 years old living in Serbia, with gender differences (*n*=314)

Food Group	Total (*n* = 314)	Women (*n* = 166)	Men (*n* = 148)	*p*
	Median (IQR)	Median (IQR)	Median (IQR)	
**Milk/milk products (kcal)**	25.2 (0.0-155.4)	27.2 (0.0-156.8)	16.3 (0.0-144.9)	0.643
**Eggs/egg products (kcal)**	0.0 (0.0-26.2)	0.0 (0.0-24.9)	0.0 (0.0-36.5)	0.721
**Meat/meat products (kcal)**	0.0 (0.0-0.0)	0.0 (0.0-0.0)	0.0 (0.0-0.0)	0.885
**Fish/seafood products (kcal)**	0.0 (0.0-0.0)	0.0 (0.0-0.0)	0.0 (0.0-0.09	0.422
**Fat/oil (kcal)**	320.2 (211.8-506.1)	285.7 (201.2-414.7)	397.7 (228.1-566.5)	**0.005****
**Grains/grain products (kcal)**	542.7 (319.1-782.7)	478.6 (303.2-677.0)	652.7 (408.9-874.1)	**<0.001*****
**Nuts/seeds/kernels (kcal)**	164.6 (57.2-318.6)	140.5 (43.8-241.7)	191.9 (66.0-471.8)	**0.004****
**Vegetables/vegetable products (kcal)**	276.2 (155.2-406.6)	231.1 (152.4-327.0)	341.5 (166.6-477.7)	**<0.001*****
**Fruits/fruit products (kcal)**	225.9 (110.7-400.4)	187.5 (103.6-327.7)	284.0 (139.4-473.1)	**0.001****
**Sugar/sweets (kcal)**	65.9 (1.8-186.8)	74.5 (6.1-180.4)	56.7 (0.5-193.1)	0.360
**Beverages/alcohol (kcal)**	41.7 (3.3-128.6)	43.7 (4.2-112.3)	28.7 (2.4-160.7)	0.958
**Miscellaneous food products (kcal)**	6.6 (2.9-11.9)	5.8 (3.1-10.8)	8.7 (2.4-16.0)	0.120
**Dietary supplements (kcal)**	0.0 (0.0-0.0)	0.0 (0.0-0.0)	0.0 (0.0-0.0)	0.873

IQR – interquartile range; *p* – statistical significance of difference (bolded values are statistically significant, ***p*<0.01, ****p*<0.001); Differences between men and women were tested with the Mann-Whitney test.

Supplementary Table S2a. Protein-related energy intake across different food groups in a sample of vegetarians/semi-vegetarians living in Serbia, 18-74 years old, with gender differences (*n*=314)

Food Group	Total (*n* = 314)	Women (*n* = 166)	Men (*n* = 148)	*p*
	Median (IQR)	Median (IQR)	Median (IQR)	
**Milk/milk products (protein kcal)**	3.9 (0.0-34.1)	5.8 (0.0-34.1)	2.8 (0.0-35.9)	0.706
**Eggs/egg products (protein kcal)**	0.0 (0.0-9.8)	0.0 (0.0-8.9)	0.0 (0.0-12.9)	0.719
**Meat/meat products (protein kcal)**	0.0 (0.0-0.0)	0.0 (0.0-0.0)	0.0 (0.0-0.0)	0.885
**Fish/seafood products (protein kcal)**	0.0 (0.0-0.0)	0.0 (0.0-0.0)	0.0 (0.0-0.0)	0.431
**Fat/oil (protein kcal)**	0.0 (0.0-0.0)	0.0 (0.0-0.0)	0.0 (0.0-0.0)	0.279
**Grains/grain products (protein kcal)**	63.1 (37.1-88.6)	54.9 (33.4-84.7)	74.1 (48.0-102.4)	**<0.001*** **
**Nuts/seeds/kernels (protein kcal)**	21.2 (7.0-3.0)	18.7 (5.7-36.0)	24.9 (8.7-58.1)	**0.011***
**Vegetables/vegetable products (protein kcal)**	56.7 (32.1-91.2)	46.2 (30.9-79.7)	68.0 (34.6-109.7)	**<0.001*** **
**Fruits/fruit products (protein kcal)**	10.1 (4.8-17.6)	9.0 (4.3-16.0)	10.6 (6.2-20.5)	**0.016****
**Sugar/sweets (protein kcal)**	0.3 (0.0-8.5)	1.0 (0.0-8.8)	0.2 (0.0-7.9)	0.291
**Beverages/alcohol (protein kcal)**	1.5 (0.0-6.1)	1.7 (0.0-6.3)	1.2 (0.0-5.6)	0.447
**Miscellaneous food products (protein kcal)**	0.9 (0.3-1.9)	0.8 (0.4-1.5)	1.1 (0.3-2.3)	0.172
**Dietary supplements (protein kcal)**	0.0 (0.0-0.0)	0.0 (0.0-0.0)	0.0 (0.0-0.0)	0.431

IQR – interquartile range; *p* – statistical significance of difference (bolded values are statistically significant, *$p<0.05$, **$p<0.01$, ***$p<0.001$); Differences between men and women were tested with the Mann-Whitney test. To convert results from kcal to g, divide by 4.

Supplementary Table S2b. Protein contribution to total energy intake across different food groups in a sample of vegetarians/semi-vegetarians living in Serbia, 18-74 years old, with gender differences (*n*=314)

Food Group	Total (*n* = 314)	Women (*n* = 166)	Men (*n* = 148)	*p*
	Median (IQR)	Median (IQR)	Median (IQR)	
**Milk/milk products (protein %TE)**	0.2 (0.0-1.7)	0.3 (0.0-1.9)	0.1 (0.0-1.5)	0.221
**Eggs/egg products (protein %TE)**	0.0 (0.0-0.4)	0.0 (0.0-0.5)	0.0 (0.0-0.4)	0.873
**Meat/meat products (protein %TE)**	0.0 (0.0-0.0)	0.0 (0.0-0.0)	0.0 (0.0-0.0)	0.880
**Fish/seafood products (protein %TE)**	0.0 (0.0-0.0)	0.0 (0.0-0.0)	0.0 (0.0-0.0)	0.416
**Fat/oil (protein %TE)**	0.0 (0.0-0.0)	0.0 (0.0-0.0)	0.0 (0.0-0.0)	0.206
**Grains/grain products (protein %TE)**	3.1 (2.1-4.0)	3.1 (2.2-4.1)	3.0 (1.9-4.0)	0.469
**Nuts/seeds/kernels (protein %TE)**	1.0 (0.4-2.1)	1.0 (0.3-2.1)	1.1 (0.4-2.1)	0.826
**Vegetables/vegetable products (protein %TE)**	2.6 (1.6-4.4)	2.5 (1.7-4.4)	2.7 (1.4-4.6)	0.938
**Fruits/fruit products (protein %TE)**	0.5 (0.3-0.9)	0.5 (0.3-0.9)	0.4 (0.3-0.9)	0.667
**Sugar/sweets (protein %TE)**	0.0 (0.0-0.4)	0.1 (0.0-0.5)	0.0 (0.0-0.3)	0.077
**Beverages/alcohol (protein %TE)**	0.1 (0.0-0.3)	0.1 (0.0-0.4)	0.1 (0.0-0.2)	0.079
**Miscellaneous food products (protein %TE)**	0.0 (0.0-0.1)	0.0 (0.0-0.1)	0.0 (0.0-0.1)	0.490
**Dietary supplements (protein %TE)**	0.0 (0.0-0.0)	0.0 (0.0-0.0)	0.0 (0.0-0.0)	0.442

%TE – percentage of total energy; IQR – interquartile range; *p* – statistical significance of difference; Differences between men and women were tested with the Mann-Whitney test.

Supplementary Table S3a. Fat-related energy intake across different food groups in a sample of vegetarians/semi-vegetarians living in Serbia, 18-74 years old, with gender differences (n=314)

Food Group	Total (n = 314)	Women (n = 166)	Men (n = 148)	p
	Median (IQR)	Median (IQR)	Median (IQR)	
Milk/milk products (fat kcal)	11.1 (0.0-88.8)	12.9 (0.0-92.8)	6.2 (0.0-79.9)	0.626
Eggs/egg products (fat kcal)	0.0 (0.0-16.5)	0.0 (0.0-15.7)	0.0 (0.0-22.9)	0.732
Meat/meat products (fat kcal)	0.0 (0.0-0.0)	0.0 (0.0-0.0)	0.0 (0.0-0.0)	0.885
Fish/seafood products (fat kcal)	0.0 (0.0-0.0)	0.0 (0.0-0.0)	0.0 (0.0-0.0)	0.412
Fat/oil (fat kcal)	314.3 (211.8-501.0)	284.7 (200.4-414.7)	394.5 (228.1-555.2)	**0.005****
Grains/grain products (fat kcal)	46.7 (26.3-83.9)	45.1 (24.5-74.7)	50.0 (27.6-97.4)	0.101
Nuts/seeds/kernels (fat kcal)	121.0 (31.7-242.5)	98.3 (26.0-172.6)	143.9 (35.8-334.4)	**0.004****
Vegetables/vegetable products (fat kcal)	26.7 (12.3-59.6)	23.9 (11.5-52.1)	30.2 (15.6-72.7)	**0.026***
Fruits/fruit products (fat kcal)	11.5 (4.9-23.1)	10.2 (4.8-21.8)	12.4 (5.1-23.5)	0.416
Sugar/sweets (fat kcal)	0.1 (0.0-56.7)	2.5 (0.0-57.1)	0.0 (0.0-55.3)	0.675
Beverages/alcohol (fat kcal)	0.7 (0.0-8.9)	2.2 (0.0-10.6)	0.2 (0.0-5.4)	**0.049***
Miscellaneous food products (fat kcal)	0.7 (0.2-1.4)	0.6 (0.3-1.2)	0.8 (0.2-1.7)	0.137
Dietary supplements (fat kcal)	0.0 (0.0-0.0)	0.0 (0.0-0.0)	0.0 (0.0-0.0)	0.059

IQR – interquartile range; p – statistical significance of difference (bolded values are statistically significant, *$p<0.05$, **$p<0.01$); Differences between men and women were tested with the Mann-Whitney test. To convert results from kcal to g, divide by 9.

Supplementary Table S3b. Fat contribution to total energy intake across different food groups in a sample of vegetarians/semi-vegetarians living in Serbia, 18-74 years old, with gender differences (*n*=314)

Food Group	Total (*n* = 314)	Women (*n* = 166)	Men (*n* = 148)	*p*
	Median (IQR)	Median (IQR)	Median (IQR)	
**Milk/milk products (fat %TE)**	0.5 (0.0-4.1)	0.7 (0.0-5.2)	0.3 (0.0-3.7)	0.217
**Eggs/egg products (fat %TE)**	0.0 (0.0-0.8)	0.0 (0.0-0.8)	0.0 (0.0-0.8)	0.922
**Meat/meat products (fat %TE)**	0.0 (0.0-0.0)	0.0 (0.0-0.0)	0.0 (0.0-0.0)	0.880
**Fish/seafood products (fat %TE)**	0.0 (0.0-0.0)	0.0 (0.0-0.0)	0.0 (0.0-0.0)	0.395
**Fat/oil (fat %TE)**	15.8 (11.2-21.9)	17.3 (12.4-21.9)	15.4 (10.4-22.2)	0.072
**Grains/grain products (fat %TE)**	2.3 (1.2-3.8)	2.5 (1.3-3.8)	2.0 (1.0-3.9)	0.162
**Nuts/seeds/kernels (fat %TE)**	5.9 (1.4-11.9)	5.6 (1.4-10.7)	6.2 (1.6-12.1)	0.480
**Vegetables/vegetable products (fat %TE)**	1.3 (0.6-2.9)	1.3 (0.6-2.7)	1.3 (0.6-2.9)	0.773
**Fruits/fruit products (fat %TE)**	0.6 (0.2-1.2)	0.7 (0.3-1.2)	0.5 (0.2-0.9)	0.180
**Sugar/sweets (fat %TE)**	0.0 (0.0-2.8)	0.1 (0.0-3.5)	0.0 (0.0-2.2)	0.267
**Beverages/alcohol (fat %TE)**	0.0 (0.0-0.5)	0.1 (0.0-0.6)	0.0 (0.0-0.2)	**0.006****
**Miscellaneous food products (fat %TE)**	0.0 (0.0-0.1)	0.0 (0.0-0.1)	0.0 (0.0-0.1)	0.525
**Dietary supplements (fat %TE)**	0.0 (0.0-0.0)	0.0 (0.0-0.0)	0.0 (0.0-0.0)	**0.032***

%TE – percentage of total energy; IQR – interquartile range; *p* – statistical significance of difference (bolded values are statistically significant, *p<0.05, **p<0.01); Differences between men and women were tested with the Mann-Whitney test.

Supplementary Table S4a. Carbohydrate-related energy intake across different food groups in a sample of vegetarians/semi-vegetarians living in Serbia, 18-74 years old, with gender differences (*n*=314)

Food Group	Total (*n* = 314)	Women (*n* = 166)	Men (*n* = 148)	*p*
	Median (IQR)	Median (IQR)	Median (IQR)	
**Milk/milk products (carbohydrate kcal)**	2.5 (0.0-18.5)	3.4 (0.0-20.9)	0.9 (0.0-14.6)	0.147
**Eggs/egg products (carbohydrate kcal)**	0.0 (0.0-0.7)	0.0 (0.0-0.6)	0.0 (0.0-0.89	0.769
**Meat/meat products (carbohydrate kcal)**	0.0 (0.0-0.0)	0.0 (0.0-0.0)	0.0 (0.0-0.0)	0.597
**Fish/seafood products (carbohydrate kcal)**	0.0 (0.0-.0)	0.0 (0.0-0.0)	0.0 (0.0-0.0)	0.403
**Fat/oil (carbohydrate kcal)**	0.0 (0.0-0.2)	0.0 (0.0-0.2)	0.0 (0.0-0.1)	0.209
**Grains/grain products (carbohydrate kcal)**	394.7 (237.4-573.9)	335.4 (215.8-493.9)	467.7 (297.7-640.6)	**<0.001***
**Nuts/seeds/kernels (carbohydrate kcal)**	14.7 (6.1-28.1)	11.9 (4.2-24.1)	15.9 (7.7-33.0)	**0.012***
**Vegetables/vegetable products (carbohydrate kcal)**	154.8 (82.1-210.4)	136.1 (76.7-180.1)	185.5 (88.3-280.6)	**<0.001***
**Fruits/fruit products (carbohydrate kcal)**	184.7 (89.9-335.1)	146.9 (84.6-276.8)	238.0 (111.7-410.3)	**<0.001***
**Sugar/sweets (carbohydrate kcal)**	41.2 (1.8-112.4)	50.8 (6.1-108.4)	32.1 (0.5-115.4)	0.312
**Beverages/alcohol (carbohydrate kcal)**	19.6 (0.5-67.5)	24.7 (1.2-64.5)	13.7 (0.0-68.8)	0.410
**Miscellaneous food products (carbohydrate kcal)**	4.5 (1.8-7.9)	4.1 (2.1-7.0)	5.7 (1.5-9.7)	0.108
**Dietary supplements (carbohydrate kcal)**	0.0 (0.0-0.0)	0.0 (0.0-0.0)	0.0 (0.0-0.0)	0.572

IQR – interquartile range; *p* – statistical significance of difference (bolded values are statistically significant, **p*<0.05, ****p*<0.001); Differences between men and women were tested with the Mann-Whitney test. To convert results from kcal to g, divide by 4.

Supplementary Table S4b. Carbohydrate contribution to total energy intake across different food groups in a sample of vegetarians/semi-vegetarians living in Serbia, 18-74 years old, with gender differences (n=314)

Food Group	Total (n = 314)	Women (n = 166)	Men (n = 148)	p
	Median (IQR)	Median (IQR)	Median (IQR)	
**Milk/milk products (carbohydrate %TE)**	0.1 (0.0-0.9)	0.2 (0.0-1.0)	0.1 (0.0-0.6)	**0.028***
**Eggs/egg products (carbohydrate %TE)**	0.0 (0.0-0.0)	0.0 (0.0-0.0)	0.0 (0.0-0.0)	0.807
**Meat/meat products (carbohydrate %TE)**	0.0 (0.0-0.0)	0.0 (0.0-0.0)	0.0 (0.0-0.0)	0.592
**Fish/seafood products (carbohydrate %TE)**	0.0 (0.0-0.0)	0.0 (0.0-0.0)	0.0 (0.0-0.0)	0.406
**Fat/oil (carbohydrate %TE)**	0.0 (0.0-0.0)	0.0 (0.0-0.0)	0.0 (0.0-0.0)	0.105
**Grains/grain products (carbohydrate %TE)**	19.4 (13.3-24.7)	19.1 (14.2-24.8)	19.5 (12.2-24.5)	0.535
**Nuts/seeds/kernels (carbohydrate %TE)**	0.7 (0.3-1.4)	0.7 (0.2-1.4)	0.7 (0.3-1.4)	0.971
**Vegetables/vegetable products (carbohydrate %TE)**	7.1 (4.2-10.6)	7.3 (4.5-10.3)	7.0 (4.0-11.2)	0.973
**Fruits/fruit products (carbohydrate %TE)**	9.7 (4.5-15.8)	9.4 (4.2-15.6)	10.0 (4.8-17.7)	0.553
**Sugar/sweets (carbohydrate %TE)**	1.9 (0.1-5.4)	2.7 (0.5-5.6)	1.3 (0.0-4.5)	**0.015***
**Beverages/alcohol (carbohydrate %TE)**	1.0 (0.0-3.3)	1.2 (0.1-4.0)	0.6 (0.0-2.8)	0.060
**Miscellaneous food products (carbohydrate %TE)**	0.2 (0.1-0.4)	0.2 (0.1-0.4)	0.2 (0.1-0.4)	0.274
**Dietary supplements (carbohydrate %TE)**	0.0 (0.0-0.0)	0.0 (0.0-0.0)	0.0 (0.0-0.0)	0.585

%TE – percentage of total energy; IQR – interquartile range; p – statistical significance of difference (bolded values are statistically significant, *p<0.05); Differences between men and women were tested with the Mann-Whitney test.

Supplementary Table S5. Comparison between different vegetarian/semi-vegetarian dietary patterns in total daily energy intake from different food groups in a sample of vegetarians/semi-vegetarians 18-74 years old, living in Serbia (*n*=314)

**Food Group**	**Vegan (*n* = 63)**	**Lacto-Ovo Vegetarian (*n* =192)**	**Semi-Vegetarian (*n* = 59)**	***p***	**Post Hoc *p***		
	**Median (IQR)**	**Median (IQR)**	**Median (IQR)**		**V vs. LOV**	**V vs. SV**	**LOV vs. SV**
**Milk/milk products (kcal)**	0.0 (0.0-0.0)	54.7 (0.0-200.8)	63.9 (0.0-189.2)	**<0.001*** **	**<0.001*** **	**<0.001*** **	1.000
**Eggs/egg products (kcal)**	0.0 (0.0-0.0)	9.5 (0.0-45.7)	0.0 (0.0-32.3)	**<0.001*** **	**<0.001*** **	**<0.001*** **	0.613
**Meat/meat products (kcal)**	0.0 (0.0-0.0)	0.0 (0.0-0.0)	0.0 (0.0-0.0)	**<0.001*** **	1.000	**<0.001*** **	**<0.001*** **
**Fish/seafood products (kcal)**	0.0 (0.0-0.0)	0.0 (0.0-0.0)	0.0 (0.0-33.3)	**<0.001*** **	1.000	**<0.001*** **	**<0.001*** **
**Fat/oil (kcal)**	267.1 (130.4-421.6)	340.7 (223.0-525.9)	346.8 (245.3-508.5)	**0.004** **	**0.009** **	**0.009** **	1.000
**Grains/grain products (kcal)**	509.6 (224.0-752.9)	572.9 (342.3-818.6)	478.4 (317.5-700.5)	0.116			
**Nuts/seeds/kernels (kcal)**	195.9 (45.2-438.4)	162.4 (57.9-289.5)	163.4 (60.7-301.9)	0.565			
**Vegetables/vegetable products (kcal)**	302.5 (180.8-478.8)	281.3 (154.7-406.7)	213.9 (131.9-380.0)	0.124			
**Fruits/fruit products (kcal)**	374.3 (157.3-642.9)	212.2 (108.4-325.0)	212.4 (107.0-412.3)	**0.001** **	**<0.001*** **	**0.050***	1.000
**Sugar/sweets (kcal)**	0.8 (0.0-86.2)	85.1 (14.4-230.0)	56.7 (7.6-141.9)	**<0.001*** **	**<0.001*** **	**0.009** **	0.360
**Beverages/alcohol (kcal)**	18.1 (0.0-122.5)	43.9 (6.2-137.6)	39.8 (3.5-116.0)	0.098			
**Miscellaneous food products (kcal)**	5.3 (1.1-14.4)	6.6 (2.9-11.9)	8.5 (3.9-13.6)	0.165			
**Dietary supplements (kcal)**	0.0 (0.0-0.0)	0.0 (0.0-0.0)	0.0 (0.0-0.0)	0.175			

V – vegan; LOV – lacto-ovo vegetarian; SV – semi-vegetarian; IQR – interquartile range; *p* – statistical significance of difference (bolded values are statistically significant, **p*<0.05, ***p*<0.01, ****p*<0.001); Differences between different dietary patterns were tested with the Kruskal-Wallis test with the post-hoc Dunn's test adjusted by the Bonferroni correction for multiple tests.

Supplementary Table S6a. Comparison between different vegetarian/semi-vegetarian dietary patterns in protein-related energy intake from different food groups in a sample of vegetarians/semi-vegetarians 18-74 years old, living in Serbia (*n*=314)

Food Group	Vegan (*n* = 63)	Lacto-Ovo vegetarian (*n* =192)	Semi-vegetarian (*n* = 59)	*p*	Post Hoc *p*		
	Median (IQR)	Median (IQR)	Median (IQR)		V vs. LOV	V vs. SV	LOV vs. SV
**Milk/milk products (protein kcal)**	0.0 (0.0-0.0)	13.2 (0.0-43.8)	13.8 (0.0-53.3)	**<0.001*****	**<0.001*****	**<0.001*****	1.000
**Eggs/egg products (protein kcal)**	0.0 (0.0-0.0)	3.7 (0.0-16.2)	0.0 (0.0-11.5)	**<0.001*****	**<0.001*****	**<0.001*****	0.651
**Meat/meat products (protein kcal)**	0.0 (0.0-0.0)	0.0 (0.0-0.0)	0.0 (0.0-0.0)	**<0.001*****	1.000	**<0.001*****	**<0.001*****
**Fish/seafood products (protein kcal)**	0.0 (0.0-0.0)	0.0 (0.0-0.0)	0.0 (0.0-13.5)	**<0.001*****	1.000	**<0.001*****	**<0.001*****
**Fat/oil (protein kcal)**	0.0 (0.0-0.0)	0.0 (0.0-0.0)	0.0 (0.0-0.1)	**0.009****	**0.012***	**0.030***	1.000
**Grains/grain products (protein kcal)**	58.2 (21.9-88.6)	67.5 (42.0-89.0)	56.0 (35.8-86.7)	0.150			
**Nuts/seeds/kernels (protein kcal)**	22.9 (8.0-53.0)	19.3 (6.8-40.9)	22.7 (7.9-42.9)	0.780			
**Vegetables/vegetable products (protein kcal)**	65.9 (34.8-97.9)	60.1 (33.1-92.6)	46.8 (27.9-73.7)	0.080			
**Fruits/fruit products (protein kcal)**	15.7 (5.4-26.8)	9.2 (4.8-15.6)	9.8 (4.4-17.2)	**0.004****	**0.003****	0.052	1.000
**Sugar/sweets (protein kcal)**	0.0 (0.0-3.2)	2.0 (0.0-12.1)	0.5 (0.0-7.0)	**<0.001*****	**<0.001*****	**0.018***	0.887
**Beverages/alcohol (protein kcal)**	0.0 (0.0-4.2)	1.8 (0.2-7.0)	1.6 (0.2-7.3)	**0.009****	**0.008****	0.087	1.000
**Miscellaneous food products (protein kcal)**	0.6 (0.1-1.6)	0.9 (0.4-1.9)	1.0 (0.6-1.9)	0.105			
**Dietary supplements (protein kcal)**	0.0 (0.0-0.0)	0.0 (0.0-0.0)	0.0 (0.0-0.0)	0.345			

V – vegan; LOV – lacto-ovo vegetarian; SV – semi-vegetarian; IQR – interquartile range; *p* – statistical significance of difference (bolded values are statistically significant, *p<0.05, **p<0.01, ***p<0.001); Differences between different dietary patterns were tested with the Kruskal-Wallis test with the post hoc Dunn's test adjusted by the Bonferroni correction for multiple tests. To convert results from kcal to g, divide by 4.

Supplementary Table S6b. Comparison between different vegetarian/semi-vegetarian dietary patterns in protein-related contribution to total energy intake from different food groups in a sample of vegetarians/semi-vegetarians 18-74 years old, living in Serbia (*n*=314)

**Food Group**	**Vegan (*n* = 63)**	**Lacto-Ovo vegetarian (*n* =192)**	**Semi-vegetarian (*n* = 59)**	***p***	**Post Hoc *p***		
	**Median (IQR)**	**Median (IQR)**	**Median (IQR)**		**V vs. LOV**	**V vs. SV**	**LOV vs. SV**
**Milk/milk products (protein %TE)**	0.0 (0.0-0.0)	0.6 (0.0-2.2)	0.7 (0.0-2.7)	**<0.001*** **	**<0.001*** **	**<0.001*** **	1.000
**Eggs/egg products (protein %TE)**	0.0 (0.0-0.0)	0.2 (0.0-0.8)	0.0 (0.0-0.6)	**<0.001*** **	**<0.001*** **	**<0.001*** **	0.647
**Meat/meat products (protein %TE)**	0.0 (0.0-0.0)	0.0 (0.0-0.0)	0.0 (0.0-0.0)	**<0.001*** **	1.000	**<0.001*** **	**<0.001*** **
**Fish/seafood products (protein %TE)**	0.0 (0.0-0.0)	0.0 (0.0-0.0)	0.0 (0.0-0.6)	**<0.001*** **	1.000	**<0.001*** **	**<0.001*** **
**Fat/oil (protein %TE)**	0.0 (0.0-0.0)	0.0 (0.0-0.0)	0.0 (0.0-0.0)	**0.016***	**0.019***	0.052	1.000
**Grains/grain products (protein %TE)**	2.7 (1.2-4.2)	3.2 (2.3-4.1)	2.9 (2.2-3.8)	0.142			
**Nuts/seeds/kernels (protein %TE)**	1.1 (0.4-2.5	0.9 (0.4-1.9)	1.2 (0.3-2.0)	0.534			
**Vegetables/vegetable products (protein %TE)**	3.2 (1.6-5.2)	2.6 (1.6-4.6)	2.2 (1.3-3.7)	**0.038***	**0.188**	**0.034***	0.638
**Fruits/fruit products (protein %TE)**	0.8 (0.3-1.5)	0.5 (0.3-0.7)	0.4 (0.2-0.9)	**<0.001*** **	1.000	**<0.001*** **	**0.030***
**Sugar/sweets (protein %TE)**	0.0 (0.0-0.1)	0.1 (0.0-0.6)	0.0 (0.0-0.4)	**<0.001*** **	**0.016***	**<0.001*** **	0.989
**Beverages/alcohol (protein %TE)**	0.0 (0.0-0.3)	0.1 (0.0-0.3)	0.1 (0.0-0.3)	**0.012***	0.113	**0.010***	1.000
**Miscellaneous food products (protein %TE)**	0.0 (0.0--0.1)	0.0 (0.0-0.1)	0.0 (0.0-0.1)	0.139			
**Dietary supplements (protein %TE)**	0.0 (0.0-0.0)	0.0 (0.0-0.0)	0.0 (0.0-0.0)	0.351			

%TE – percentage of total energy; V – vegan; LOV – lacto-ovo vegetarian; SV – semi-vegetarian; IQR – interquartile range; *p* – statistical significance of difference (bolded values are statistically significant, **p*<0.05, ****p*<0.001); Differences between different dietary patterns were tested with the Kruskal-Wallis test with the post hoc Dunn's test adjusted by the Bonferroni correction for multiple tests. To convert results from kcal to g, divide by 4.

Supplementary Table S7a. Comparison between different vegetarian/semi-vegetarian dietary patterns in fat-related energy intake from different food groups in a sample of vegetarians/semi-vegetarians 18-74 years old, living in Serbia (*n*=314)

Food Group	Vegan (*n* = 63)	Lacto-Ovo vegetarian (*n* =192)	Semi-vegetarian (*n* = 59)	*p*	Post Hoc *p*		
	Median (IQR)	Median (IQR)	Median (IQR)		V vs. LOV	V vs. SV	LOV vs. SV
**Milk/milk products (fat kcal)**	0.0 (0.0-0.0)	28.5 (0.0-110.3)	38.3 (0.0-127.1)	**<0.001*****	**<0.001*****	**<0.001*****	1.000
**Eggs/egg products (fat kcal)**	0.0 (0.0-0.0)	5.1 (0.0-28.7)	0.0 (0.0-20.3)	**<0.001*****	**<0.001*****	**<0.001*****	0.627
**Meat/meat products (fat kcal)**	0.0 (0.0-0.0)	0.0 (0.0-0.0)	0.0 (0.0-0.0)	**<0.001*****	1.000	**<0.001*****	**<0.001*****
**Fish/seafood products (fat kcal)**	0.0 (0.0-0.0)	0.0 (0.0-0.0)	0.0 (0.0-11.8)	**<0.001*****	1.000	**<0.001*****	**<0.001*****
**Fat/oil (fat kcal)**	263.1 (127.2-421.6)	328.1 (223.0-517.6)	345.4 (245.3-508.5)	**0.005****	**0.011***	**0.008****	1.000
**Grains/grain products (fat kcal)**	50.8 (13.5-101.6)	49.4 (26.9-84.2)	40.0 (29.9-56.7)	0.263			
**Nuts/seeds/kernels (fat kcal)**	145.0 (22.8-306.9)	116.0 (31.7-214.6)	118.2 (36.3-212.9)	0.527			
**Vegetables/vegetable products (fat kcal)**	26.6 (13.9-49.3)	31.9 (12.4-68.1)	17.5 (10.5-45.1)	0.085			
**Fruits/fruit products (fat kcal)**	17.7 (6.0-42.2)	10.7 (5.0-20.5)	11.0 (4.5-19.0)	0.079			
**Sugar/sweets (fat kcal)**	0.0 (0.0-16.2)	18.1 (0.0-78.7)	0.0 (0.0-49.7)	**<0.001*****	**<0.001*****	0.182	0.426
**Beverages/alcohol (fat kcal)**	0.0 (0.0-6.3)	1.2 (0.0-10.9)	1.4 (0.0-12.2)	0.061			
**Miscellaneous food products (fat kcal)**	0.6 (0.0-1.3)	0.6 (0.3-1.3)	0.8 (0.4-1.7)	0.166			
**Dietary supplements (fat kcal)**	0.0 (0.0-0.0)	0.0 (0.0-0.0)	0.0 (0.0-0.0)	0.479			

V – vegan; LOV – lacto-ovo vegetarian; SV – semi-vegetarian; IQR – interquartile range; *p* – statistical significance of difference (bolded values are statistically significant, **p*<0.05, ***p*<0.01, ****p*<0.001); Differences between different dietary patterns were tested with the Kruskal-Wallis test with the post hoc Dunn's test adjusted by the Bonferroni correction for multiple tests. To convert results from kcal to g, divide by 9.

Supplementary Table S7b. Comparison between different vegetarian/semi-vegetarian dietary patterns in fat-related contribution to total energy intake from different food groups in a sample of vegetarians/semi-vegetarians 18-74 years old, living in Serbia (n=314)

Food Group	Vegan (*n* = 63)	Lacto-Ovo vegetarian (*n* =192)	Semi-vegetarian (*n* = 59)	*p*	Post Hoc *p*		
	Median (IQR)	Median (IQR)	Median (IQR)		V vs. LOV	V vs. SV	LOV vs. SV
**Milk/milk products (fat %TE)**	0.0 (0.0-0.0)	.6 (0.0-5.4)	1.9 (0.0-7.0)	**<0.001*** **	**<0.001*** **	**<0.001*** **	1.000
**Eggs/egg products (fat %TE)**	0.0 (0.0-0.0)	0.3 (0.0-1.3)	0.0 (0.0-1.0)	**<0.001*** **	**<0.001*** **	**<0.001*** **	0.585
**Meat/meat products (fat %TE)**	0.0 (0.0-0.0)	0.0 (0.0-0.0)	0.0 (0.0-0.0)	**<0.001*** **	1.000	**<0.001*** **	**<0.001*** **
**Fish/seafood products (fat %TE)**	0.0 (0.0-0.0)	0.0 (0.0-0.0)	0.0 (0.0-0.6)	**<0.001*** **	1.000	**<0.001*** **	**<0.001*** **
**Fat/oil (fat %TE)**	13.2 (7.3-20.9)	15.9 (11.7-21.4	17.8 (12.8-23.7)	**0.005** **	**0.035* **	**0.005** **	0.498
**Grains/grain products (fat %TE)**	2.2 (0.6-4.6)	2.3 (1.3-3.8)	2.1 (1.2-3.1)	0.505			
**Nuts/seeds/kernels (fat %TE)**	8.2 (0.9-13.7)	5.5 (1.5-9.5)	5.9 (1.3-9.8)	0.319			
**Vegetables/vegetable products (fat %TE)**	1.2 (0.7-2.3)	1.3 (0.6-3.3)	1.0 (0.5-1.7)	0.097			
**Fruits/fruit products (fat %TE)**	0.8 (0.3-2.0)	0.5 (0.2-0.9)	0.6 (0.2-1.1)	**0.027* **	1.000	**0.025* **	0.130
**Sugar/sweets (fat %TE)**	0.0 (0.0-0.8)	0.7 (0.0-3.6)	0.0 (0.0-2.1)	**<0.001** **	0.188	**<0.001*** **	0.477
**Beverages/alcohol (fat %TE)**	0.0 (0.0-0.3)	0.1 (0.0-0.5)	0.1 (0.0-0.6)	0.091			
**Miscellaneous food products (fat %TE)**	0.0 (0.0-0.1)	0.0 (0.0-0.1)	0.0 (0.0-0.1)	0.178			
**Dietary supplements (fat %TE)**	0.0 (0.0-0.0)	0.0 (0.0-0.0)	0.0 (0.0-0.0)	0.482			

%TE – percentage of total energy; V – vegan; LOV – lacto-ovo vegetarian; SV – semi-vegetarian; IQR – interquartile range; *p* – statistical significance of difference (bolded values are statistically significant, *p<0.05, **p<0.01, ***p<0.001); Differences between different dietary patterns were tested with the Kruskal-Wallis test with the post hoc Dunn's test adjusted by the Bonferroni correction for multiple tests.

Supplementary Table S8a. Comparison between different vegetarian/semi-vegetarian dietary patterns in carbohydrate-related energy intake from different food groups in a sample of vegetarians/semi-vegetarians 18-74 years old, living in Serbia (*n*=314)

Food Group	Vegan (*n* = 63)	Lacto-Ovo Vegetarian (*n* =192)	Semi-Vegetarian (*n* = 59)	*p*	Post Hoc *p*		
	Median (IQR)	Median (IQR)	Median (IQR)		V vs. LOV	V vs. SV	LOV vs. SV
**Milk/milk products (carbohydrates kcal)**	0.0 (0.0-0.0)	8.4 (0.0-22.5)	4.9 (0.0-24.0)	**<0.001*** **	**<0.001*** **	**<0.001*** **	1.000
**Eggs/egg products (carbohydrate kcal)**	0.0 (0.0-0.0)	0.2 (0.0-1.0)	0.0 (0.0-0.7)	**<0.001*** **	**<0.001*** **	**<0.001*** **	0.634
**Meat/meat products (carbohydrate kcal)**	0.0 (0.0-0.0)	0.0 (0.0-0.0)	0.0 (0.0-0.0)	**<0.001*** **	1.000	**<0.001*** **	**<0.001*** **
**Fish/seafood products (carbohydrate kcal)**	0.0 (0.0-0.0)	0.0 (0.0-0.0)	0.0 (0.0-0.0)	**<0.001*** **	1.000	**<0.001*** **	**<0.001*** **
**Fat/oil (carbohydrate kcal)**	0.0 (0.0-0.1)	0.0 (0.0-0.2)	0.0 (0.0-0.2)	0.174			
**Grains/grain products (carbohydrate kcal)**	375.6 (158.1-510.1)	436.7 (257.7-591.2)	368.9 (236.2-553.2)	0.071			
**Nuts/seeds/kernels (carbohydrate kcal)**	14.4 (6.2-33.0)	14.3 (5.9-25.5)	16.3 (5.8-38.1)	0.801			
**Vegetables/vegetable products (carbohydrate kcal)**	175.3 (103.8-250.5)	154.8 (80.5-205.6)	134.1 (68.6-210.3)	0.110			
**Fruits/fruit products (carbohydrate kcal)**	305.4 (121.0-566.4)	169.4 (87.3-278.5)	180.7 (95.3-346.1)	**<0.001*** **	**<0.001*** **	**0.047***	1.000
**Sugar/sweets (carbohydrate kcal)**	0.8 (0.0-50.1)	61.8 (13.6-126.5)	43.4 (7.6-90.6)	**<0.001*** **	**<0.001*** **	**0.010***	0.313
**Beverages/alcohol (carbohydrate kcal)**	12.2 (0.0-76.5)	22.5 (2.4-75.0)	17.8 (0.0-47.7)	0.128			
**Miscellaneous food products (carbohydrate kcal)**	3.7 (0.9-7.6)	4.2 (1.9-7.8)	5.3 (2.5-8.3)	0.222			
**Dietary supplements (carbohydrate kcal)**	0.0 (0.0-0.0)	0.0 (0.0-0.0)	0.0 (0.0-0.0)	0.087			

V – vegan; LOV – lacto-ovo vegetarian; SV – semi-vegetarian; IQR – interquartile range; *p* – statistical significance of difference (bolded values are statistically significant, *p<0.05, ***p<0.001); Differences between different dietary patterns were tested with the Kruskal-Wallis test with the post hoc Dunn's test adjusted by the Bonferroni correction for multiple tests. To convert results from kcal to g, divide by 4.

Supplementary Table S8b. Comparison between different vegetarian/semi-vegetarian dietary patterns in carbohydrate-related contribution to total energy intake from different food groups in a sample of vegetarians/semi-vegetarians 18-74 years old, living in Serbia (n=314)

Food Group	Vegan (n = 63)	Lacto-Ovo Vegetarian (n =192)	Semi-Vegetarian (n = 59)	p	Post Hoc p		
	Median (IQR)	Median (IQR)	Median (IQR)		V vs. LOV	V vs. SV	LOV vs. SV
**Milk/milk products (carbohydrate %TE)**	0.0 (0.0-0.0)	0.4 (0.0-1.0)	0.2 (0.0-0.9)	**<0.001*****	**<0.001*****	**<0.001*****	1.000
**Eggs/egg products (carbohydrate %TE)**	0.0 (0.0-0.0)	0.0 (0.0-0.1)	0.0 (0.0-0.0)	**<0.001*****	**<0.001*****	**<0.001*****	0.623
**Meat/meat products (carbohydrate %TE)**	0.0 (0.0-0.0)	0.0 (0.0-0.0)	0.0 (0.0-0.0)	**<0.001*****	1.000	**<0.001*****	**<0.001*****
**Fish/seafood products (carbohydrate %TE)**	0.0 (0.0-0.0)	0.0 (0.0-0.0)	0.0 (0.0-0.0)	**<0.001*****	1.000	**<0.001*****	**<0.001*****
**Fat/oil (carbohydrate %TE)**	0.0 (0.0-0.0)	0.0 (0.0-0.0)	0.0 (0.0-0.0)	0.200			
**Grains/grain products (carbohydrate %TE)**	16.1 (7.3-25.1)	20.5 (14.7-25.2)	18.4 (14.2-23.7)	0.065			
**Nuts/seeds/kernels (carbohydrate %TE)**	0.7 (0.2-1.7)	0.6 (0.3-1.3)	0.8 (0.3-1.5)	0.578			
**Vegetables/vegetable products (carbohydrate %TE)**	8.5 (5.7-12.9)	6.9 (4.1-10.3)	6.2 (3.4-9.3)	**0.010***	0.983	**0.013***	**0.033***
**Fruits/fruit products (carbohydrate %TE)**	14.9 (5.5-33.5)	8.5 (4.3-13.6)	8.8 (3.7-18.1)	**<0.001*****	0.790	**<0.001*****	**0.028***
**Sugar/sweets (carbohydrate %TE)**	0.0 (0.0-2.7)	2.8 (0.6-6.2)	2.0 (0.4-4.3)	**<0.001*****	**0.012***	**<0.001*****	0.288
**Beverages/alcohol (carbohydrate %TE)**	0.5 (0.0-4.1)	1.0 (0.1-3.8)	0.8 (0.0-2.2)	0.114			
**Miscellaneous food products (carbohydrate %TE)**	0.2 (0.1-0.3)	0.2 (0.1-0.4)	0.3 (0.1-0.4)	0.174			
**Dietary supplements (carbohydrate %TE)**	0.0 (0.0-0.0)	0.0 (0.0-0.0)	0.0 (0.0-0.0)	0.088			

%TE – percentage of total energy; V – vegan; LOV – lacto-ovo vegetarian; SV – semi-vegetarian; IQR – interquartile range; p – statistical significance of difference (bolded values are statistically significant, *p<0.05, ***p<0.001); Differences between different dietary patterns were tested with the Kruskal-Wallis test with the post hoc Dunn's test adjusted by the Bonferroni correction for multiple tests.
